# Accelerated Discovery of Halide Perovskite Materials via Computational Methods: A Review

**DOI:** 10.3390/nano14131167

**Published:** 2024-07-08

**Authors:** Ming Sheng, Hui Zhu, Suqin Wang, Zhuang Liu, Guangtao Zhou

**Affiliations:** 1 College of Engineering, Shandong Xiehe University, Jinan 250109, China; shengming@sdxiehe.edu.cn (M.S.); zhuhui@sdxiehe.edu.cn (H.Z.); wangsuqin@sdxiehe.edu.cn (S.W.); 2 Key Laboratory for Liquid-Solid Structural Evolution and Processing of Materials, Ministry of Education, Shandong University, Jinan 250061, China

**Keywords:** halide perovskites, computational methods, high-throughput screening

## Abstract

Halide perovskites have gained considerable attention in materials science due to their exceptional optoelectronic properties, including high absorption coefficients, excellent charge-carrier mobilities, and tunable band gaps, which make them highly promising for applications in photovoltaics, light-emitting diodes, synapses, and other optoelectronic devices. However, challenges such as long-term stability and lead toxicity hinder large-scale commercialization. Computational methods have become essential in this field, providing insights into material properties, enabling the efficient screening of large chemical spaces, and accelerating discovery processes through high-throughput screening and machine learning techniques. This review further discusses the role of computational tools in the accelerated discovery of high-performance halide perovskite materials, like the double perovskites A_2_BX_6_ and A_2_BB′X_6_, zero-dimensional perovskite A_3_B_2_X_9_, and novel halide perovskite ABX_6_. This review provides significant insights into how computational methods have accelerated the discovery of high-performance halide perovskite. Challenges and future perspectives are also presented to stimulate further research progress.

## 1. Introduction

Halide perovskites have captured considerable interest in materials science due to their exceptional optoelectronic properties, which include high absorption coefficients, outstanding charge-carrier mobilities, and adjustable band gaps [1]. These attributes render them highly promising for applications in photovoltaics, light-emitting diodes, synaptic devices, and other optoelectronic technologies [2,3,4].

Pursuing high-performance halide perovskites involves optimizing their stability, efficiency, and manufacturability. At present, while a power conversion efficiency (PCE) of above 25% in a laboratory-scale device is being achieved by most of the leading laboratories [5], long-term stability still stands as the main formidable obstacle for large-scale commercialization, as it exhibits structural instability when exposed to moisture and electric fields, presenting additional challenges in their development [5]. In addition, lead toxicity remains another profound concern [6,7].

To address these issues, researchers increasingly focus on discovering stable, lead-free alternatives that can offer similar or superior performance. Key performance metrics include high PCE, long-term operational stability, and the ability to process materials cheaply and with scalable techniques. Achieving these goals requires a deep understanding of the materials’ properties and their ability to predict how changes in compositions and structures will impact performance. This quest necessitates the exploration of vast compositional and structural spaces, a task that is somehow impractical through traditional experimental methods due to the associated time and cost. Moreover, halide perovskite materials are notable for their versatile structures, both in terms of their inorganic framework and overall shape [8]. For example, their structural flexibility allows them to form low-dimensional versions, which are more resistant to moisture and have adjustable crystal sizes [9,10,11]. Thus, a deeper understanding of the mechanism of the structure–activity relationship is greatly needed for the design of high-performance halide perovskites [12].

Thus, computational methods have emerged as essential tools for the design and discovery of new halide perovskite materials. Firstly, they enhance our understanding of material properties. For instance, they provide insights into the stability and reaction mechanisms of halide perovskites, elucidating how various factors like ionic radii, electronic interactions, and external conditions impact their structural integrity and functionality [13,14]. For example, perovskite materials with larger bandgaps are more stable under light exposure because higher energy barriers reduce the non-ideal recombination of photo-generated carriers. With multi-scale simulation techniques like ab initio molecular dynamics (AIMD), the time and length scales of a material’s behavior can be dynamically revealed and can be used to understand the structural evolution during a reaction [15]. Moreover, computational methods enable the efficient screening of large chemical spaces, significantly accelerating the discovery process, so-called high-throughput screening. Consequently, significant efforts are being dedicated to developing new materials with improved stability and optimal performance [4,5,16]. Recent advancements in machine learning have played a pivotal role in this process by enabling the direct and fast prediction of material properties from vast datasets, significantly reducing the computational cost of density functional theory (DFT) calculations, allowing researchers to explore a larger chemical space in less time [12]. Techniques such as support vector machines, random forests, and deep learning models like graph neural networks have been used [17]. Some typical examples include Lu et al. for identifying the lead-free halide perovskites [18] and Sun et al. for the discovery of novel A_3_B_2_X_9_ perovskites [16].

This review sets the stage for discussing the accelerated discovery of halide perovskite materials through computational tools that are widely utilized and highlighting their application towards high-performance halide perovskites. The computational tools can boost understanding of the structure–activity relationship by considering the structural stability and electronic structure. Moreover, computational methods enable the high-throughput screening of large chemical spaces, significantly accelerating the discovery process. We then elaborate on the significant roles of computational methods in accelerating the discovery of some significant halide perovskites like the double perovskites A_2_BX_6_ and A_2_BB′X_6_ and novel halide perovskite ABX_6_. Looking ahead, the continued development and integration of advanced computational techniques for machine learning hold great promise for unlocking new, high-performance materials with tailored properties for a wide range of applications.

## 2. Computational Tools

### 2.1. Structural Stability

Predicting the structural stability of halide perovskites remains a significant challenge in the discovery of new functional materials. The formation energy is a critical measure for evaluating the stability and synthesizability of halide perovskites. This energy can be defined in two main ways: (1) in relation to the constituent unary, binary, or multinary phases or (2) in comparison to the stable linear combination of competing phases in the phase diagram (E_hull_) [19].

Generally, the above energy can be calculated via DFT methods. However, they are more focused on the ground-state properties at zero Kelvin and hugely ignore the stability under different working conditions [12]. Effectively bridging time and length scales is essential for the discovery of halide perovskites, as many dynamic properties of interest cannot be directly obtained from static DFT calculations, and this requires multi-scale strategies [15]. In this case, ab initio thermodynamics (AITD) can be utilized to assess the temperature-dependent stabilities under a varying condition. Raman et al. [20] investigate the exsolution of Pt from ATiO_3_ perovskites (where A = Ca, Sr, or Ba) under different temperatures, which finally suggests that Pt exsolution is influenced by the formation of sub-surface vacancies, followed by the diffusion of Pt to the surface, and this process depends significantly on the specific host perovskite and its exposed facets. To further consider the time-dependent stabilities, ab initio molecular dynamic simulations can be performed to indicate whether a halide perovskite can maintain its structural integrity at a random temperature [18]. Such a technique is more focused on a system’s dynamics and statistical properties [12]. Moreover, the solvent effect can be also considered explicitly in the simulation. In some cases, the solvent can destabilize the perovskite [21].

For perovskites, some reliable empirical factors have been applied to evaluate the structural stability. Through simple calculation, one can assess the stability of perovskite materials quickly, which saves a lot of time compared to DFT simulations. Among them, the ionic radii play a crucial role in shaping our understanding of bonding and stability in perovskites. For example, Goldschmidt’s tolerance factor (t) has been widely employed [13,14] as follows:

$$t=\left(r_{A}+r_{X}\right)/\sqrt{2}\left(r_{B}+r_{X}\right)$$

Here, *r*_i_ (i = A, B, or X) represents the radius of a specific ion in perovskites. The calculated tolerance factors of typical perovskite materials range between 0.81 and 1.11 [22]. Predicting the stability of perovskites using the tolerance factor requires only their chemical compositions.

Furthermore, when the octahedral index (*μ*), the following is true:

$$\mu =r_{B}/r_{X}$$

This factor falls within the range of 0.41 to 0.90, a significant distortion of the octahedral geometry is suggested, and there is a high likelihood of multiphase coexistence [13]. The ionic radii, tolerance factor, and octahedral factor have been regarded as the most important criteria for classification, indicating that steric and geometric packing effects are key to the stability of halide perovskites [17].

With the help of the machine learning (ML) techniques, the new tolerance factor (t) can be discovered. Bartel et al. [23] present a new tolerance factor (*τ*) using an SISSO algorithm, which has the following form:

$$\tau =\frac{r_{X}}{r_{B}}-n_{A}\left(n_{A}-\frac{r_{A}/r_{B}}{\ln(r_{A}/r_{B})}\right)$$

where $n_{A}$ is the oxidation state of A, *r*_i_ is the ionic radius of ion i, $r_{A}\;$>$\;r_{B}$ by definition, and *τ* < 4.18 is the perovskite. A high overall accuracy and generalizability were achieved for an overly broad range of halide perovskites.

When it comes to the stability of halide perovskites, structural distortions are significant. These distortions impact the crystal structure by altering bond lengths, angles, and overall lattice symmetry to minimize the system’s energy. Structural distortions in perovskite materials occur due to various factors such as differences in ionic radii, electronic interactions like the Jahn–Teller effect, temperature changes causing phase transitions, external mechanical stress, chemical doping, and the presence of defects. The tolerance factor serves as an indicator of the degree of distortion from an idealized cubic structure. Typically, a stabilized cubic structure will have a relatively large tolerance factor, greater than 0.9 [24]. Moreover, the DFT calculations can also help to confirm distortion in the perovskites, such as the existence of sublattice distortion in Na-doped Cs_2_AgBiCl_6_ double perovskites [25]. The more dynamic distortion processes in different environments like temperature can be revealed via AITD or AIMD [26].

### 2.2. Electronic Structure

Electronic structure analysis is extremely important in the research of halide perovskites because their unique electronic structure properties determine their optoelectronic and electronic performance to a large extent. The calculated electronic structure can also be used to predict the absorption spectrum, using which researchers can gain detailed insights into how perovskite absorbs light at different wavelengths [5,27,28]. In addition, the calculated electronic structure provides detailed insights into the stability. By examining the electronic structure, researchers can identify features such as antibonding orbitals, which are molecular orbitals where electron occupation weakens or disrupts chemical bonds, reducing material stability [29], or identify defect states (e.g., vacancies and interstitial atoms) [30].

Among the different properties of electronic structures, bandgap is a crucial parameter that significantly impacts their performances. While the efficiency of a realistic device depends on various properties, the bandgap is widely used as the screening criterion due to its convenience in DFT calculations [15]. Firstly, the bandgap is related to the stability and durability of halide perovskites. Perovskite materials with larger bandgaps are more stable under light exposure because higher energy barriers reduce the non-ideal recombination of photo-generated carriers. Moreover, the size of the bandgap directly affects the photovoltaic conversion efficiency. Perovskite solar cells with low bandgaps can absorb a broader spectrum of sunlight, enhancing their efficiency and overall effectiveness in photovoltaic applications [31]. Materials with the bandgaps larger than 2.0 eV are typically unsuitable for photovoltaics [3]. For example, if a multijunction solar cell consists of multiple materials with different bandgaps, its overall photovoltaic conversion efficiency can be enhanced as each absorbs different wavelengths of light. In this case, perovskite materials can be tuned to match the bandgaps of other materials such as silicon or copper indium gallium selenide, creating efficient multijunction solar cells. It is worth noting that a direct bandgap allows electrons to directly emit photons when transitioning from the conduction band to the valence band, while an indirect bandgap requires an additional phonon interaction for this transition, which can be studied differently in different halide perovskite systems.

The electronic structure of a perovskite can also influence its reactivity. For instance, the electronic structure and the oxidation state of the B cation are predicted to impact the reactivity of perovskites in relation to dissociative oxygen adsorption and the formation energies of oxygen vacancies [32]. For example, perovskites with mixed cations at the B site, maintaining a 3Pb:2Sn molar ratio, have demonstrated promising efficiency, reaching 15.1% [33].

### 2.3. Rational Design and High-Throughput Screening

Improvements in computational capacity, along with better simulation algorithms, now make it possible to accurately calculate the properties of many materials efficiently. This means we can explore a lot more materials quickly, which boosts our understanding of their properties and potential applications. Consequently, there is a growing emphasis on high-throughput screening of the vast chemical space, which greatly enhances our understanding and intuition regarding material properties. For instance, hundreds of halide perovskites have been screened using DFT and high-throughput calculations, leading to the discovery of high-performance materials. Several candidates, such as Cs_2_BB′X_6_ (where B = In, Tl; B′ = Sb, Bi; X = Cl, Br), have emerged as promising photovoltaic absorber materials [5].

Machine learning plays a pivotal role in this process by enabling the direct and fast prediction of material properties from vast datasets, significantly reducing the computational cost of DFT calculations, and allowing researchers to explore a larger chemical space in less time. In discovering and studying halide perovskite materials, various ML techniques can be applied. These include, but are not limited to, the following: traditional ML methods based on feature engineering such as support vector machines (SVMs) and random forests for predicting material properties and structural stability [17]. Deep learning techniques such as graph neural networks (GNNs) are used for the efficient processing of large-scale structural data and predicting material performance [34]. In many material-based ML studies, predicting the stability of materials (formation energies, energy above hull) is often the primary goal, or at least a significant subgoal, alongside key application-specific metrics such as ionic conductivity, catalytic adsorption energies and activities, bandgaps, etc. For example, Schmidt et al. [35] developed crystal graph attention networks with a mean absolute error (MAE) of 30 meV/atom, which are then applied to identify several thousand potentially stable ABCD_2_ compounds through the high-throughput search of 15 million candidates based on the predicted distance to the convex hull. Takahashi et al. [36] employed a ML model to categorize the bandgap of perovskite materials using 18 physical descriptors, which finally identify 10 thermodynamically stable undiscovered perovskite materials with ideal band gaps for solar light capturing from 414736 perovskite data items.

## 3. Accelerated Discovery of Novel Halide Perovskites

In this section, we classify emerging halide perovskites into several groups according to their different spatial configurations and elemental compositions, and the significant roles that computational methods play in accelerating the prediction of these systems are then elaborated.

### 3.1. Vacancy-Ordered Double Perovskites: A_2_BX_6_

**Figure 1:**
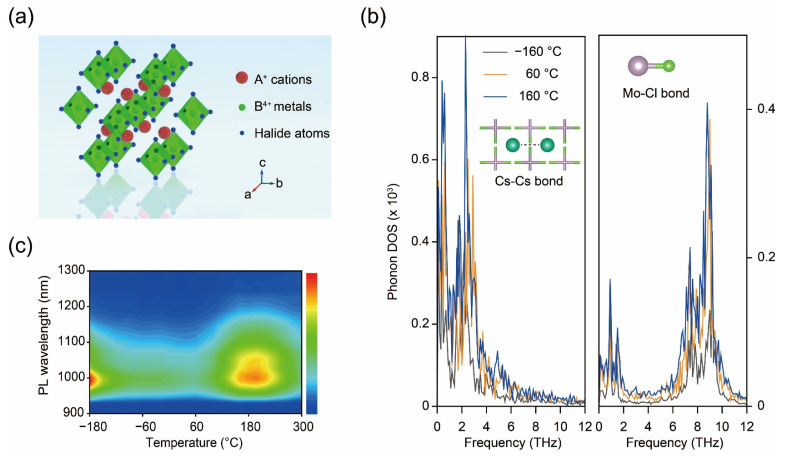
(**a**) Atomic structure of zero-dimensional A_2_BX_6_ halide perovskites. (**b**) Projected phonon densities of states for the Cs-Cs bond (left) and Mo-Cl bond (right) as a function of temperature. (**c**) Photoluminescence spectra of Cs_2_MoCl_6_ as temperature varies. Reprinted with permission from Ref. [52]. Copyright 2022, Wiley.

A_2_BX_6_-typed zero-dimensional (0D) halide perovskite materials, known as vacancy-ordered double perovskites, or perovskite variants, are a newly emerging class of novel compounds that have garnered rapid research attention in recent years [9,10,11]. Compared to their double perovskite cousins, they maintain a 0D electronic structure, i.e., all octahedra do not have contact with each other directly to form a plane or line, with all tetravalent metal ions located at the octahedral centers [37]. Six halide anions bind with a one-metal cation to form an octahedron, separated by the monovalent A-site constituents (Figure 1a). The independently dispersed octahedra throughout the entire lattice endow the crystal structure with periodically missing fundamental units, therefore imparting unique properties to this material category [38,39,40]. DFT calculations have been comprehensively utilized to predict the performances of perovskite variants [41,42,43,44,45]. Compared to other categories of perovskite materials, the most remarkable advantages of perovskite variants are the ease of forming self-trapping excitons (STEs) and exceptional chemical stability [46,47,48].

Unlike bandgap emission excitons, STEs are transient excited states. In another word, when the external irradiation is removed, STEs disappear immediately [49]. The creation of such states is greatly facilitated in perovskite variants due to their low-dimensional electronic structures, which result in localized charge distribution and strong carrier–phonon coupling [50]. In addition, owing to their lower energy levels, STEs generally emit light with large Stokes shifts and longer wavelengths [51]. In a recent study, researchers conducted DFT calculations to predict two new types of perovskite variants Cs_2_MoCl_6_ and Cs_2_WCl_6_, capable of emitting near-infrared (NIR) light [52]. The computational results indicate that the novel perovskite variants composed of heavy metals exhibit more pronounced octahedra distortions, i.e., Jahn–Teller distortion, under photoexcitation processes, which is the so-called heavy metal effect [26]. In addition, perturbed octahedra become more notable with increasing temperature, as evidenced by the temperature-dependent projected phonon density of states, conducted via AIMD simulations (Figure 1b). As the temperature elevates, Cs-Cs and Mo-Cl bonds are largely extended, suggesting strengthened lattice distortion, and facilitate formation of STEs.

As predicted by DFT calculations, the photoluminescence (PL) spectra of Cs_2_MoCl_6_ crystals exhibit a broadband emission extending into the NIR-II region, marking the longest observed radiation wavelength in perovskite hosts to date (Figure 1c). The significant Stokes shift of 550–600 nm, coupled with a broadband emission spanning 200–300 nm, indicates STE emission, which is commonly observed in low-dimensional halide perovskites, particularly those containing heavy metals [53,54]. Moreover, like other 0D halide perovskites, these crystals do not display photoluminescence based on free excitons, likely due to the rapid generation of trapped excitons caused by structural distortion. More importantly, in the high-temperature region, the increase in emission intensity indicates the temperature-facilitated formation of STEs and the thermally activated release of trapped charge carriers, demonstrating the good alignment of experimental results with theoretical calculations [55].

**Figure 2:**
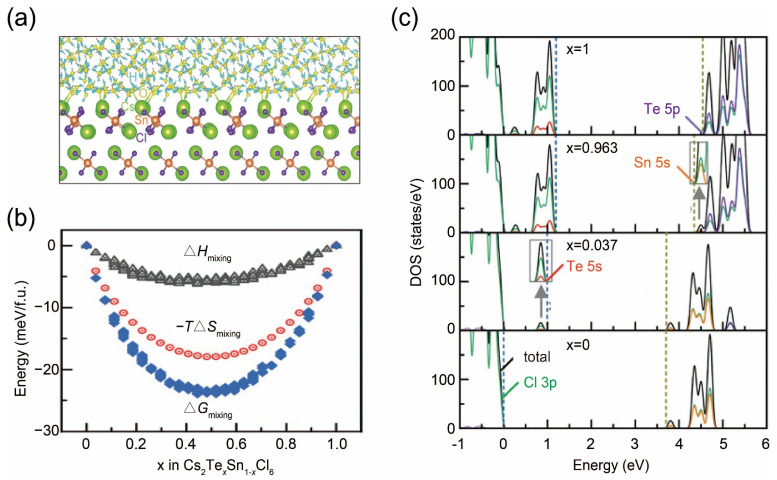
(**a**) DFT-simulated structure of the water/Cs_2_SnCl_6_ interface. H, O, Sb, Cl, and Cs atoms are marked as light pink, red, gray, light green, and drak green, respectively. (**b**) The calculated Gibbs free energy (Δ*G*_mixing_) for mixing Sn and Te at the B sites to form Cs_2_Sn_1−x_Te_x_Cl_6_ solid solutions, along with the enthalpic (Δ*H*_mixing_) and entropic (−*T*Δ*S*_mixing_) contributions, presented as functions of *x*. (**c**) Calculated total and projected densities of states for Cs_2_Sn_1−x_Te_x_Cl_6_. Reprinted with permission from Ref. [21]. Copyright 2020, Wiley.

The instability of halide perovskite poses a significant obstacle to their practical applications [56,57]. In a recent study, molecular dynamics (MD) simulations were performed to investigate the anti-water stability of the perovskite variant Cs_2_SnCl_6_ for its interface with water [21]. Calculation results indicate that the surface terminated by specific bonds shows the lowest surface energy and, thus, the best stability, and no reaction occurs at the water/Cs_2_SnCl_6_ interface (Figure 2a). Considering the low photoluminescence efficiency of Cs_2_SnCl_6_, researchers compute the feasibility of Te doping strategies and conduct DFT calculations to gain more insights into the electronic structures of Cs_2_(Te_x_Sn_1−x_)Cl_6_ solid solutions. The calculated Gibbs free energy of mixing metals, along with the corresponding enthalpic and entropic contributions as functions of the doping ratio, demonstrates that the solid solution reaction is a thermodynamically favorable and spontaneous process (Figure 2b). More importantly, DFT calculations predict the formation of long-pair ns^2^ electrons with Te doping. Unlike Sn^4+^, Te^4+^ behaves filled pseudo-closed 5s^2^ orbitals, leading to a higher absorption coefficient and extended absorption window (Figure 2c) [58]. In addition, ns^2^ electronic configuration suggests an antibonding characteristic at the valence-band maximum, facilitating the formation of shallow-level acceptor defects with low formation energies, rather than deep-level defects that act as traps and recombination centers, which endow them with higher defect tolerance and improved stability [29]. Based on aforementioned calculation prediction, the perovskite variant Cs_2_(Te_x_Sn_1−x_)Cl_6_ is synthesized using a facile hydrothermal approach, and as-prepared samples show a pretty high photoluminescence quantum yield (PLQY) of 95.4% and exceptional anti-water stability, which is consistent with the calculation results.

**Figure 3:**
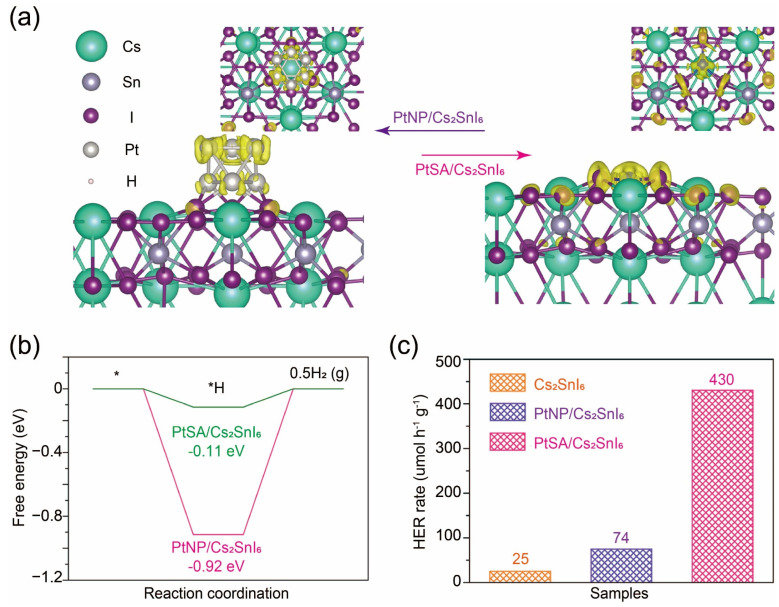
(**a**) Charge density difference maps before and after photoexcitation for PtNP/Cs_2_SnI_6_ (left) and PtSA/Cs_2_SnI_6_ (right). (**b**) The calculated energy profile for hydrogen production on PtNP/Cs_2_SnI_6_and PtSA/Cs_2_SnI_6_. * stands for the hydrogen adsorption process. (**c**) The rate of photocatalytic hydrogen evolution over PtSA/Cs_2_SnI_6_, PtNP/Cs_2_SnI_6_, and Cs_2_SnI_6_. Reprinted with permission from Ref. [28]. Copyright 2021, The Nature Publishing Group.

Although halide perovskite materials have found utility in optical and optoelectronic fields, the potential uses of halide perovskite materials in other application domains are still rarely explored. Considering their inherent ability for facile composition modification, it is imperative to explore and develop novel halide perovskite materials with specific compositions. In this regard, computational methods demonstrate excellent performances in predicting other properties of perovskite variants and their respective application domains [59,60,61]. In a recent study, DFT calculation was used to evaluate the photogenerated charge transfer on PtSA/Cs_2_SnI_6_ and its analogue PtNP/Cs_2_SnI_6_, showing that Pt nanoparticle is anchored on Cs_2_SnI_6_ (Figure 3a) [28]. The difference in charge density before and after photoexcitation indicates that photogenerated electrons disperse throughout the entire Pt nanoparticle in PtSA/Cs_2_SnI_6_, leading to a reduction in electron density per Pt atom within the nanoparticle. In contrast, in PtSA/Cs_2_SnI_6_, electrons are predominantly localized between PtSA and the neighboring three I atoms, resulting in high electron density at the Pt-I3 site. Consequently, PtNP exhibits a lower electron density per Pt atom compared to PtSA, indicating the relatively electron-deficient nature of PtNP. This suggests that PtSA has a stronger capability to capture electrons from Cs_2_SnI_6_, resulting in higher hydrogen production activity. Therefore, the distinct electronic properties of PtNP and PtSA on Cs_2_SnI_6_ contribute to their differing catalytic dynamics in the hydrogen evolution reaction (HER), with PtSA showing a significantly lower energy barrier than PtNP (Figure 3b). Based on simulation results, researchers designed and synthesized PtSA/Cs_2_SnI_6_ complexes and their counterparts as control groups to investigate their photocatalytic HER performances. The photocatalytic activities for hydrogen evolution over PtSA/Cs_2_SnI_6_, PtNP/Cs_2_SnI_6_, and Cs_2_SnI_6_ catalysts were assessed in an aqueous solution of hydroiodic acid under identical conditions (Figure 3c). PtSA/Cs_2_SnI_6_ exhibits superior activity for photocatalytic hydrogen production, which is 17.2 and 5.8 times higher than those of pristine Cs_2_SnI_6_ and optimized PtNP/Cs_2_SnI_6_, respectively.

### 3.2. Traditional Double Perovskites: A_2_BB′X_6_

**Figure 4:**
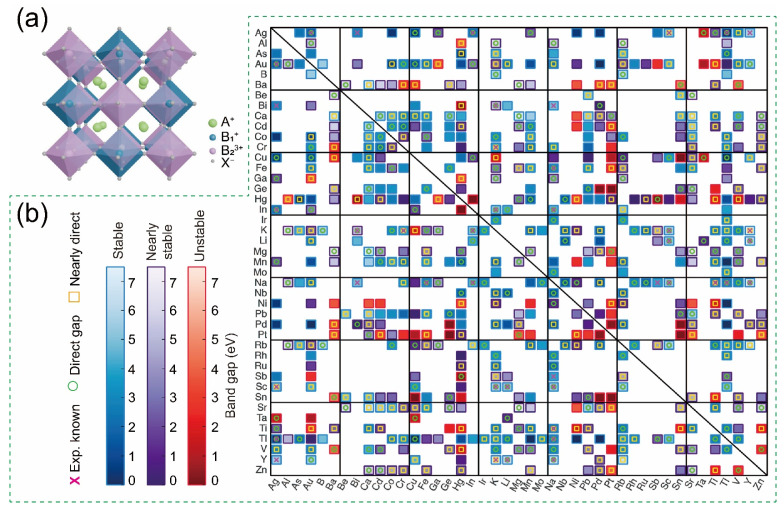
(**a**) The typical crystal structure of double perovskite materials. (**b**) Map illustrating the properties of Cs_2_BB′Cl_6_, with B and B′ defined along each axis. Reprinted with permission from Ref. [66]. Copyright 2020, The American Chemical Society.

Given the great harm of toxicity in lead-based halide perovskites, lead-free perovskites, which offer lower toxicity and enhanced stability as alternative materials, have become a central research focus in recent years [62,63]. Early research primarily focused on Sn^2+^ and Ge^2+^ cations as substitutes for Pb^2+^, but these halide perovskites are even more unstable, mainly due to the easy oxidation of these elements in ambient conditions [64,65]. Another strategy involves replacing every two Pb^2+^ cations with one monovalent cation and one trivalent cation, creating a charge-ordered double perovskite with the general formula A_2_BB′X_6_ (A and B = monovalent cations; B′ = trivalent cations; X = halogen anions), known as double perovskites (Figure 4a). Compared to the vacancy-ordered double perovskites discussed in Section 3.1, traditional double perovskites possess quite different structures, even though they both hold a 0D configuration. In detail, vacancy-ordered double perovskites periodically lose octahedra, while traditional double perovskites maintain a complete octahedral construction. To date, double perovskites have shown promise as high-performance materials in various applications, including electrocatalysts, ferroelectrics, white light emitters, phosphors, magneto-resistive materials, and spintronics [66].

Considering the multitude of potential components for double perovskites, researchers have employed efficient simulation methods to predict easily synthesizable compositions and anticipate their various properties [3,66,67]. In a representative study, researchers selected 311 double perovskites, Cs_2_B^1^B^2^Cl_6_, from a pool of 903 compounds as potentially stable candidates based on a statistically learned tolerance factor for perovskite stability. These 311 double perovskites then underwent DFT calculations to evaluate their stability and identify candidates with suitable band gaps for optoelectronic applications (Figure 4b). Thermodynamic analysis predicted that 261 of these 311 compounds are likely to be synthesized, as they are stable against decomposition into competing compounds. Among these 261 candidates, 47 are free of toxic elements and exhibit direct or nearly direct band gaps within a desirable range for photovoltaic applications, as confirmed by DFT calculations. Notably, a specific subgroup, Cs_2_[Alk]^+^[TM]^3+^Cl_6_, where Alk represents group 1 alkali atoms and TM denotes a transition-metal cation, forms a category of Cs_2_BB′Cl_6_ double perovskites characterized by large and tunable exciton binding energies.

**Figure 5:**
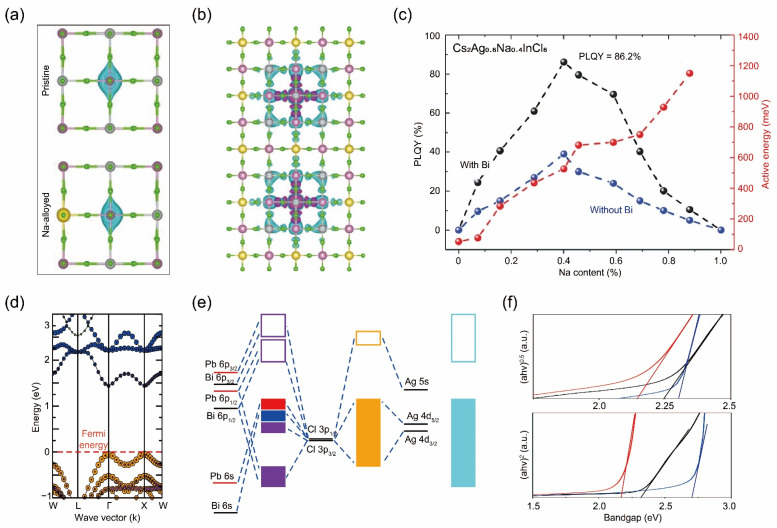
(**a**) Change in the parity of the electron wavefunction of STEs before and after Na incorporation. Reprinted with permission from Ref. [68]. Copyright 2018, The Nature Publishing Group. (**b**) Configuration illustrating the reinforced confinement of the STEs within two lattice parameters, surrounded by the NaCl6 octahedra. (**c**) Activation energy and PLQY of Cs2AgxNa1-xInCl6 samples as a function of sodium content. (**d**) Band structure of Cs_2_(Pb^I^Pb^III^)_0.25_(AgBi)_0.75_Br_0.75_Cl_6_. (**e**) Molecular orbital scheme for Pb-doped Cs2AgBiCl6. (**f**) Tauc plots from the absorption spectra of Cs_2_(Pb^I^Pb^III^)_0.25_(AgBi)_0.75_Br_0.75_Cl_6_. Reprinted with permission from Ref. [27]. Copyright 2018, The American Physical Society.

Another representative work utilizing computational methods involved analyzing the electronic structures of double perovskites. By incorporating Na cations into Cs_2_AgInCl_6_, researchers were able to disrupt the inversion-symmetry-induced parity-forbidden transition, also known as the dark transition. This was achieved by manipulating the parity of the wavefunction of the self-trapped excitons and decreasing the electronic dimensionality, resulting in a three-order-of-magnitude increase in photoluminescence efficiency compared to pristine samples [25,68]. Computational results showed that the incorporation of Na disrupts the inversion symmetry of the Cs_2_AgInCl_6_ lattice, altering the electron wavefunction at the Ag site from symmetric to asymmetric (Figure 5a). This change in parity in the self-trapped excitons’ wavefunction enables radiative recombination. Additionally, since Na^+^ does not contribute to either the conduction-band minimum or the valence-band maximum, its incorporation decreases the electronic dimensionality of the Cs_2_AgInCl_6_ lattice by partially isolating the [AgCl_6_]^5−^ octahedra [36]. Consequently, the [NaCl_6_]^5−^ octahedra in the alloyed compounds act as barriers, restricting the spatial distribution of the self-trapped excitons, enhancing the overlap between electron and hole orbitals, and increasing the transition dipole moment (Figure 5b). As a result, the optimally composed Bi-doped Cs_2_(Ag_0.60_Na_0.40_)InCl_6_ emits warm-white light with a quantum efficiency of 86% and operates continuously for over 1000 h (Figure 5c) [68].

A very recent study looked at a novel type of double perovskite Cs_2_(Pb^I^Pb^III^)_0.25_(B_1_^I^B_2_^III^)_0.75_X_6_ (X = Cl, Br, I), in which the same metal element with different oxidation states partially replaced the central metal atoms in the octahedral sites, significantly affecting the electronic structure of the original perovskite matrix [27]. We refer to this doping strategy as a form of disproportionate doping. Before Pb doping, pristine Cs_2_AgBiCl_6_ shows an indirect bandgap [69,70,71]. However, Pb substitution alters the topology of the band structure, inducing an indirect-to-direct transition that is optically allowed (Figure 5d). It also significantly reduces the bandgap, leading to absorption in visible wavelengths. Additional calculations on the optical absorption spectra of these disproportionated compounds reveal absorption coefficients and solar efficiencies that are comparable to or even higher than those of the state-of-the-art photovoltaic absorber material, CH_3_NH_3_PbI_3_ [72]. To gain a deeper understanding of the bandgap structure, a qualitative molecular orbital diagram for Cs_2_(Pb^I^Pb^III^)_0.25_(B_1_^I^B_2_^III^)_0.75_X_6_ was drawn, along with the orbital-projected density of states. The results show that spin–orbit coupling plays a pivotal role in shaping the electronic structures of these systems. The incorporation of spin–orbit coupling induces a splitting of the Bi and Pb p orbitals, leading to a reduction in the conduction band primarily at the Γ and X points, which is the main cause of the transition from an indirect to a direct bandgap (Figure 5e). Additionally, transitioning the halide from Cl to Br and then to I elevates the valence-band maximum due to the increase in atomic p-orbital energy associated with decreasing electronegativity [73]. The valence bandwidth diminishes as the energy disparity between B-d and X-p orbitals narrows. The enhanced delocalization of p orbitals as the halide shifts from Cl to I expands the bottom of the conduction band, resulting in a reduced bandgap from chlorides to iodides [74,75].

To verify the simulation results, the experimental characterization of Cs_2_(Pb^I^Pb^III^)_0.25_(AgBi)_0.75_Br_6_ was conducted. The measured properties, including bandgap and chemical stability, align closely with theoretical predictions. For instance, the absorption spectra exhibited a sharp onset at approximately 550 nm for Cs_2_AgBiBr_6_, while a redshift of 30 nm was noted for Cs_2_(Pb^I^Pb^III^)_0.25_(AgBi)_0.75_Br_6_. From the Tauc plot results, direct bandgaps of 2.31 eV and 2.13 eV were determined, respectively (Figure 5f). Their respective indirect bandgaps were measured at 2.21 eV and 2.10 eV. The Pb^2+^ substitution resulted in a reduction in the bandgap for both parent compounds. Notably, although the indirect bandgaps for the parent compounds are significantly lower than their respective direct bandgaps, the direct and indirect bandgaps of the Pb-doped compound are nearly identical. This observation corroborates the theoretical findings, suggesting a transition from an indirect to a direct bandgap. It is worth mentioning that Cs_2_(Pb^I^Pb^III^)_0.25_(AgBi)_0.75_Br_6_ has demonstrated superior stability compared to CH_3_NH_3_PbI_3_, even under conditions of elevated humidity and temperature [76]. Consequently, disproportionate double perovskite materials have the potential to surpass state-of-the-art materials and emerge as more promising candidates.

### 3.3. Perovskite-Related Materials: A_3_B_2_X_9_

Halide perovskites are renowned for their appeal in radiation detection due to their excellent intrinsic properties. These materials possess a large average atomic number, a high carrier mobility–charge carrier lifetime product, and high resistivity, making them ideal for high-sensitivity X-ray detection [77,78,79]. In addition, halide perovskites possess larger average atomic number, i.e., proton number (*Z*), making them promising as X-ray blocking materials. Sensitive X-ray detection is crucial in various applications, such as therapeutic and diagnostic healthcare, industrial inspection, security screening, and scientific research [80,81,82]. There are two general approaches for X-ray detection: indirect conversion using scintillators and direct conversion of X-ray photons into electronic signals. The latter approach is more advantageous due to its higher spatial resolution and simpler system configuration. Utilizing reduced X-ray dose rates is often desirable, especially for applications related to human and environmental security, emphasizing the need for highly sensitive X-ray detectors.

**Figure 6:**
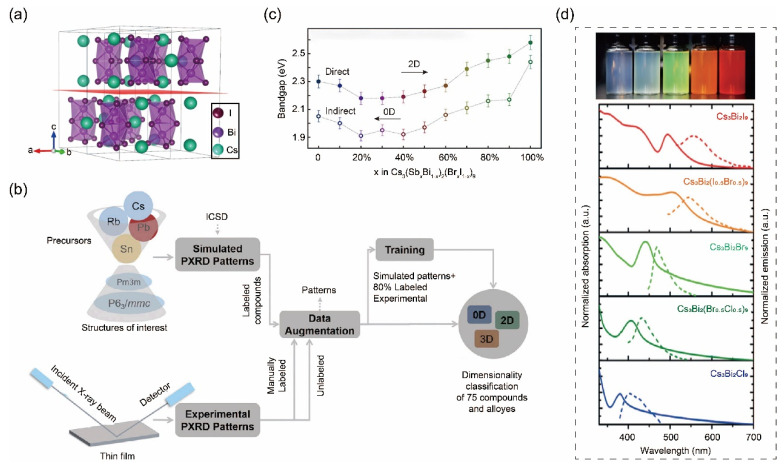
(**a**) Crystal configuration of A_3_B_2_X_9_ materials. Reprinted with permission from Ref. [93]. Copyright 2020, The Nature Publishing Group. (**b**) A schematic workflow for utilizing machine learning algorithms to aid in the structural characterization of perovskite-inspired materials and to guide the analysis of diagnostic results. Reprinted with permission from Ref. [16]. Copyright 2019, Cell Press. (**c**) Estimated band gaps of the Cs_3_(Bi_1−x_Sb_x_)_2_(I_1−x_Br_x_)_9_ alloy series for x ranging from 0 to 1. (**d**) Photographs of as-prepared colloidal Cs_3_Bi_2_X_9_ nanocrystals under UV excitation (top) and their corresponding absorption and PL spectra (bottom). Reprinted with permission from Ref. [94]. Copyright 2017, Wiley.

Recently, perovskite-related materials A_3_B_2_X_9_ (A = monovalent constituents, B = trivalent metals, X = halogen anions) have attracted considerable attention for high-energy radiation detection due to their high-density and wide-bandgap semiconducting features [83,84,85,86,87]. A_3_B_2_X_9_ compounds usually possess hexagonal zero-dimensional spatial configurations (Figure 6a). In detail, [B_2_X_9_]^3−^ dimers are formed by the face-sharing of [BX_6_]^3−^ octahedra, with A-site atoms spatially separating them to construct the zero-dimensional structure.

Considering the multitude of potential components in A_3_B_2_X_9_, high-throughput screening has been performed to screen for promising perovskite materials that could be potentially synthesized. In a recent study, researchers generated lattice structures by populating lattice positions with anions and cations selected from a list of permissible species [4]. They also generated a range of potential precursors from which a given material could potentially decompose to estimate the material’s stability. The electronic energies of both the material and the precursors were calculated to determine the formation energy of the material relative to its precursors. As a result, some components more likely to be synthesized under mild conditions were identified and later verified in experimental reports [88,89,90,91,92].

Furthermore, the application of machine learning techniques to aid in the discovery of novel A_3_B_2_X_9_ perovskites has been documented [16]. By increasing experimental throughput, researchers successfully synthesized and characterized 75 new halide perovskite compositions, comprising 17 compounds and 58 alloy compositions, with 4 compounds being reported for the first time. More intriguingly, the dual-site alloy series Cs_3_(Sb_x_Bi_1−x_)_2_(Br_x_I_1−x_) was introduced, showcasing a transition from zero-dimensional to two-dimensional structures and non-linear bandgap tunability. The discovery of non-linear bandgap behavior in this series paves the way for the development of halide perovskites for multijunction solar cells.

In practical implementation, researchers initially acquire a database rapidly through high-throughput synthesis, encompassing various experimental properties from samples with varying compositions, which serves as the testing dataset. In the meantime, simulated X-ray diffraction (XRD) patterns are utilized as the training dataset within the identical neural network architecture and hyperparameters. Data augmentation significantly improves classification accuracy, surpassing a baseline below 60% achieved with non-augmented data. However, relying exclusively on simulated XRD patterns for training captures nuanced differences among experimental XRD patterns, encompassing systematic errors in sample alignment and random human errors in synthesis. To accurately represent these experimental intricacies, collected powder XRD patterns of unique compositions are segregated into two experimental datasets. Group 1 includes manually identified perovskites with space group and dimensionality labels, while Group 2 comprises materials lacking detailed structure information (Figure 6b). To further refine accuracy, the final training dataset integrates simulated data with a randomly selected 80% subset of Group 1 experimental patterns. Through cross-validation, a blindfold model achieves 90% accuracy for Group 1 materials, highlighting substantial improvements in model accuracy and robustness by incorporating experimental data into the training set.

Through the utilization of machine learning, not only can the composition of materials be predicted, but so can the evolution of properties exhibited by the materials as the composition varies continuously. In this study, it is observed that the band gap undergoes a non-continuous transition in the alloy series Cs_3_(Bi_1−x_Sb_x_)_2_(I_1−x_Br_x_)_9_. Specifically, the alloy exhibits a band gap lower than either of its zero-dimensional or two-dimensional end phases. Machine learning diagnostics highlight that starting from around a 20% dopant level of Cs_3_Sb_2_Br_9_, the alloy begins to manifest characteristics typical of a two-dimensional (2D) perovskite crystal structure. Remarkably, alongside the structural transformation, there is a non-linear relationship observed in the optical properties, marking a novel phenomenon in lead-free perovskite-inspired materials (Figure 6c).

The aforementioned optical properties have been confirmed in subsequent reports [83,84,95,96,97,98]. For instance, Cs_3_Bi_2_X_9_ (X = Cl, Br, I) nanocrystals are synthesized through a straightforward room-temperature reaction, resulting in emission wavelengths spanning from 400 to 560 nm, corresponding to a band gap ranging from 2.2 eV to 3.1 eV, which aligns well with the calculated results (Figure 6d) [94]. In another study, a nucleation-controlled solution method for growing large, high-quality Cs_3_Bi_2_I_9_ perovskite single crystals is reported, and researchers successfully harvest centimeter-sized single crystals possessing pre-eminent X-ray detection performance with a high sensitivity of 1652 μC Gyair^−1^ cm^−2^ and exceptionally low detectable dose rate of 130 nGyair s^−1^, both of which are highly desirable for medical diagnostics [93]. These examples demonstrate that combining an accelerated experimental cycle with machine learning-based diagnostics represents a significant step towards achieving fully automated laboratories for material discovery and development.

Inspired by the aforementioned computational methods applied to perovskite-related materials such as A_3_B_2_X_9_, numerous studies have emerged in recent years. For instance, a Cs_3_Bi_2_I_9_ single-crystalline thin film was seamlessly integrated onto various substrates, including Si wafers, using a straightforward and low-temperature solution-processing technique [85]. The excellent lattice match and band alignment between Si(111) and Cs_3_Bi_2_I_9_(001) facets facilitate photo-generated charge dissociation and extraction, significantly enhancing photoelectric sensitivity by 10 to 200 times compared to photodetectors based on other substrates. In another research, through an environmentally friendly and straightforward process utilizing ethanol as the anti-solvent, the synthesized Cs_3_Bi_2_Br_9_ quantum dots exhibit blue emission at 410 nm with a PLQY of up to 19.4% [84]. Furthermore, Cs_3_Bi_2_Br_9_ quantum dots demonstrate exceptional photo-stability and moisture stability owing to their all-inorganic composition and surface passivation.

### 3.4. Novel Halide Perovskites: ABX_6_

ABX_6_ compounds have a structure where A is a monovalent atom, B is a pentavalent cation, and X is a halogen. ABX₆ compounds typically adopt a double-perovskite structure, where two BX₆ octahedra are connected through shared vertices. The A-site cations are in the gaps between the octahedra [99]. These compounds often have high symmetry, contributing to their excellent crystalline and electronic properties. Based on Goldschmidt’s tolerance factor and octahedral index, the stability of ABX_6_ depends on having appropriate ionic radii to maintain the lattice integrity.

**Figure 7:**
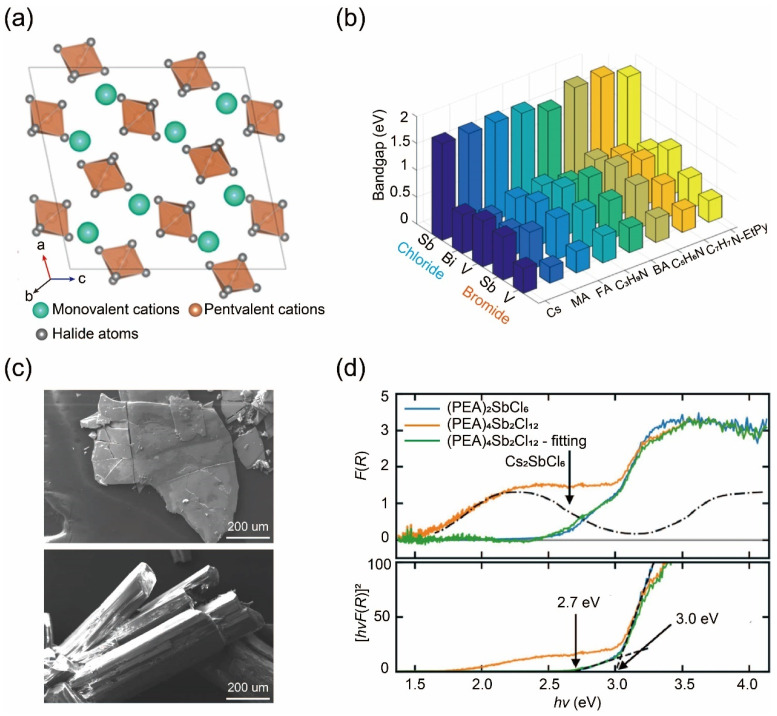
(**a**) Crystal structure of ABX5 materials. Reprinted with permission from Ref. [100]. Copyright 2018, The American Chemical Society. (**b**) Bandgaps of ABX6 series compositions computed via DFT. (**c**) SEM images of (PEA)SbCl_6_ (top) and (PEA)_4_Sb^III^Sb^V^Cl_12_ compounds (bottom). (**d**) Kubelka–Munk-transformed diffuse reflectance spectra (top) and the corresponding Tauc plots (bottom) of studied compounds. Reprinted with permission from Ref. [101]. Copyright 2021, Wiley.

The crystalline structures of ABX_6_, when viewed from the [010] and [101] directions, exhibit distinct characteristics compared to other zero-dimensional perovskite compounds (Figure 7a). When observed from the [010] direction, the octahedra [BX_6_]^−^ and cations are arranged into two distinct rows, alternating in sequence. Conversely, when viewed from the [101] direction, these components form layers composed of rows of octahedra [BX6]^−^ and cations, which stack together to create lamellar structures. Moreover, to evaluate the stability of the novel zero-dimensional materials against decomposition, researchers calculate the formation energy associated with commonly utilized synthetic routes for producing ABX_6_. It is evident that most chlorides are predicted to be thermodynamically stable, and the perovskites composed of V and Sb exhibit higher stability compared to their Bi-based counterparts. Subsequently, the electronic properties of these compounds are calculated, including bandgaps (Figure 7b). Our results show that chloride compounds generally exhibit wider bandgaps compared to bromides. Among them, Sb-chlorides have the widest bandgaps, while Sb-bromides possess wider bandgaps than V-bromides. Bi-chlorides have bandgaps like those of Sb-based bromides. It is worth noting that the size of the cation A^+^ can significantly influence the bandgaps of the compounds, e.g., the bandgap value is 0.35 eV higher for CsSbBr_6_ than N-EtPySbBr_6_, as calculated using hybrid DFT functional.

To validate predictions regarding the structures and properties of ABX_6_ perovskites, extensive experimental efforts are focused on synthesizing such materials [102,103,104,105,106]. Exemplified by (PEA)SbCl_6_ (PEA = phenylethylammonium), results indicate that B-site metals not only exist in pentavalence but also ease the transfer to trivalence to form (PEA)_4_Sb^III^Sb^V^Cl_12_ compounds [101]. The geometries observed in SEM images vary significantly, as (PEA)SbCl_6_ exhibits a sheet-like shape, whereas (PEA)_4_Sb^III^Sb^V^Cl_12_ crystallizes into a rod-like structure (Figure 7c). The Kubelka–Munk-transformed diffuse reflectance spectra of (PEA)SbCl_6_ and (PEA)_4_Sb^III^Sb^V^Cl_12_ are provided to facilitate a comparison of their optical properties. Overall, there is a moderate level of agreement between the experimentally obtained optical bandgaps and the electronically calculated electronic bandgaps. However, it is important to note that the optical properties of the two compounds exhibit significant differences in detail (Figure 7d). The yellow hue of (PEA)SbCl_6_ indicates absorption primarily in the blue spectrum, with significant absorption extending into the near-ultraviolet range. Conversely, the spectrum for the dark red, mixed-valence (PEA)_4_Sb^III^Sb^V^Cl_12_ exhibits similarities in the ultraviolet range, and it also demonstrates weaker, more uniform absorption across the blue, green, and yellow wavelengths, gradually tapering off towards the near-infrared wavelength. This lower energy absorption is likely attributed to intervalence charge transfer between Sb^III^ and Sb^V^, supported by its resemblance to the low energy feature observed in Cs_2_SbCl_6_ [107].

In next step, the investigation of ABX_6_ perovskites needs to be focused on further discovering new properties experimentally, as predicted by computation methods. For instance, zero-dimensional perovskites based on the Group VA elements Sb and Bi exhibit characteristics akin to molecular crystals, including a comparable absorption spectrum to those of sunlight and carrier mobilities suitable for photovoltaic applications [100]. These unique properties arise from the strong hybridization between the s orbital of the Group VA element and the p orbital of the halide in ASbBr_6_ and ABiCl_6_, with significant overlap of these hybrid states among neighboring octahedra.

## 4. Conclusions and Perspectives

In summary, this review underscores the significant advancements and future potential of halide perovskite materials, especially in the context of computational methods accelerating their discovery and optimization. Computational methods are essential for gaining insights into material properties, enabling accelerated material discovery through high-throughput screening and ML techniques. The ongoing challenges, such as achieving long-term stability and addressing lead toxicity, are being actively tackled through the development of novel perovskite compositions and structures with the help of computational methods.

In addition to the four halide perovskite materials discussed in this review, computational methods have played an important role in studying other sub-categories of perovskites, such as novel antiperovskites and chalcogenide perovskites. For example, through ion-type inversion and anion ordering on perovskite lattice sites, two novel classes of pnictogen-based quaternary antiperovskites have been devised [5]. More importantly, their phase stability levels and adjustable band gaps were then comprehensively predicted through first-principle calculations in order to further screen these materials as photovoltaic candidates. Moreover, computational methods have been applied to predict the ferroelectric properties in molecular ferroelectrics, and many other intriguing electrical properties have been investigated as well [108]. Furthermore, chalcogenide perovskites, as state-of-the-art perovskite materials, have been exploited with the assistance of computational methods [109]. Overall, the integration of advanced computational methods with experimental efforts is paving the way for the next generation of high-performance halide perovskite materials.

## Data Availability

The data presented in this study are available in the article.
